# Detection of Circulating HPV16 DNA as a Biomarker for Cervical Cancer by a Bead-Based HPV Genotyping Assay

**DOI:** 10.1128/spectrum.01480-21

**Published:** 2022-02-28

**Authors:** Luisa Galati, Jean-Damien Combes, Florence Le Calvez-Kelm, Sandrine McKay-Chopin, Nathalie Forey, Mathis Ratel, James McKay, Tim Waterboer, Lea Schroeder, Gary Clifford, Massimo Tommasino, Tarik Gheit

**Affiliations:** a International Agency for Research on Cancer, Lyon, France; b Infections and Cancer Epidemiology, German Cancer Research Center (DKFZ), Heidelberg, Germany; Memorial Sloan Kettering Cancer Center

**Keywords:** E6 antibodies, HPV biomarkers, HPV16, cervical cancer, circulating HPV DNA, liquid biopsy

## Abstract

Human papillomavirus (HPV) circulating tumor DNA (HPV ctDNA) was proposed as a biomarker for the detection and disease monitoring of HPV-related cancers. One hundred eighty plasma samples obtained from women diagnosed with HPV16-positive cervical cancer (CC) (*n* = 100), HPV16-positive premalignant lesions (cervical intraepithelial neoplasia grade 3 [CIN3]) (*n* = 20), and HPV DNA-negative controls (*n* = 60) were randomly selected from the archives for evaluating the performance of a bead-based HPV genotyping assay (E7 type-specific multiplex genotyping assay [E7-MPG]) in detecting HPV16 ctDNA. The performance of the E7-MPG was compared with those of DNA detection by droplet digital PCR (ddPCR) and detection of HPV16 E6 antibodies evaluated in an independent study. Internal controls to assess DNA quality were included in the molecular assays, i.e., beta-globin and ESR1, respectively. The sensitivity and specificity of E7-MPG and/or E6 antibodies to detect HPV16-positive CCs were evaluated. HPV16 ctDNA was detected using the E7-MPG in 42.3% of all plasma samples and in 74.7% of plasma samples from HPV16-positive CC cases. The validation of E7-MPG data by ddPCR showed that the sensitivity of the E7-MPG test for HPV16-positive CC detection was higher than that of ddPCR (74.7% versus 63.1%; *P* < 0.001). When both HPV16 ctDNA and E6 antibodies were considered, the sensitivity for HPV16-positive CC detection increased from 74.7% to 86.1%, while the specificity was unchanged at 97.8%. The performance of E7-MPG for the detection of HPV16 ctDNA appears to be at least as sensitive as that of ddPCR, offering an additional tool for ctDNA detection of HPV16-positive CC. The use of an additional blood marker of HPV infection, such as E6 antibodies, further improved the detection of CC.

**IMPORTANCE** The validity of HPV ctDNA as a marker of HPV-driven cancers has been previously reported. Herein we validated an alternative to ddPCR for HPV16 ctDNA detection, using a bead-based HPV genotyping assay that offers the potential advantage of reducing the cost of clinical management due to the multiplex capability of the test, thus facilitating its use in clinical settings. In addition, we analyzed HPV ctDNA in the context of E6 antibodies as an additional HPV marker. The HPV16 ctDNA biomarker appeared to be highly specific and, to a lesser extent, sensitive for the detection of CC, mainly indicated for those at an advanced tumor stage. In this proof-of-principle study, E6 antibodies were mainly detected in early tumor stages of CC, while HPV ctDNA was mainly positive at advanced tumor stages.

## INTRODUCTION

A subgroup of mucosal human papillomaviruses (HPVs) has been clearly associated with human carcinogenesis. An International Agency for Research on Cancer (IARC) monograph classified the alpha-HPVs HPV16, -18, -31, -33, -35, -39, -45, -51, -52, -56, -58, and -59 as high-risk (HR) types, as they are carcinogenic to humans (group 1) (1). The HR-HPVs are responsible for about 4.5% of worldwide annual cancers (1), including anogenital and a subgroup of head and neck cancers (1). Despite the important achievements in the screening and prevention of HPV-associated cancers, mainly in high-income countries, cervical cancer (CC) still represents a significant fraction of virus-related cancers, accounting for 530,000 estimated new cases worldwide (2), mainly from low- and middle-income countries. In addition, the incidence of HPV-related anal and head and neck cancers is rising steadily in Europe and North America (3, 4). Guidelines for screening of HPV-associated cancers are based on cervical cytology and HPV DNA testing (5) and on anal cytology and high-resolution anoscopy for anal cancer screening in high-risk groups (e.g., HIV patients among men who have sex with men [MSM]) (6, 7). In contrast, for head and neck cancers, there is still a lack of validated screening tools for the identification of HPV-driven cancers. Several HPV markers (e.g., HPV circulating tumor DNA [HPV ctDNA] and HPV antibodies) have been evaluated for early diagnosis of different HPV-associated cancers as possible alternative adjunct tools (8–13). In the last decade, body fluids have received attention for the detection of HPV markers since they are easy to collect and handle, as well as easily transferable into clinical practice. ctDNA is released into the bloodstream from cancer cells by necrotic and/or apoptotic events or through active secretion (14). Several studies provide evidence for the validity of ctDNA as a diagnostic and prognostic biomarker of HPV-positive cancers (15–17). Since ctDNA levels appear to increase with the severity of the disease (18–20), the technical challenge of detecting early-stage lesions lies in the ability of the test to detect low levels of ctDNA. Thus, several methodologies have been applied to increase the sensitivity of the detection of ctDNA (20–24). Droplet digital PCR (ddPCR) is considered a well validated quantitative method for HPV ctDNA detection (15), although additional assays with a higher diagnostic sensitivity are still required for the early detection of malignancies. In this study, we evaluated the performance of the well-validated multiplex HPV E7 type-specific multiplex genotyping bead-based assay E7-MPG (25, 26) and compared it to that of ddPCR for the detection of HPV16 ctDNA. We analyzed plasma specimens from patients with HPV16-positive premalignant and malignant cervical lesions. We also compared HPV16 ctDNA positivity with the presence of antibodies against the viral oncogenic HPV16 E6 protein.

## RESULTS

One hundred eighty archived plasma samples (from 100 patients with cervical squamous cell carcinoma [CC], 20 patients with cervical intraepithelial neoplasia grade 3 [CIN3], and 60 controls) were analyzed for HPV16 ctDNA using the HPV type-specific multiplex genotyping assay E7-MPG. Of these 180 samples, 35 specimens (21 CC cases and 14 controls) were excluded due to insufficient DNA quality as evidenced by a negative beta-globin result, as well as three duplicate samples (2 CIN3 cases and 1 control), leaving a total of 142 samples (79 CC cases, 18 CIN3 cases, and 45 controls).

The performance of the E7-MPG assay for the detection of HPV16 ctDNA was evaluated to measure its sensitivity and specificity for the detection of HPV16-positive CCs in the complete series of 142 beta-globin-positive plasma samples, including samples from 79 HPV16-positive CC cases at different stages (T1, *n* = 17; T2, *n* = 32; T3, *n* = 25; and T4, *n* = 4; in one case, this information was not available), 18 HPV16-positive CIN3 cases, and 45 HPV-negative controls. Using the E7-MPG methodology, HPV16 ctDNA was found in 42.3% (60/142) of all plasma samples. More specifically, 74.7% (59/79) of the plasma samples from CC cases tested positive for HPV16 ctDNA, while the plasma samples from patients with premalignant lesions (CIN3) were all negative for HPV16 ctDNA (Table 1; Table S2 in the supplemental material). Among the controls, two specimens tested positive for HPV ctDNA, one with HPV16 and the other with HPV18 ctDNA (Table 1; Table S1).

**TABLE 1:** HPV16 ctDNA results by E7-MPG assay and HPV16 E6 serology data stratified in cases and controls

Assay, result	No. (%) of participants who were:							
	Controls	CIN3 cases^*a*^	Patients with cancer stage:					Total no.
			T1	T2	T3	T4	Unknown T	
HPV16 ctDNA								
Positive	1 (2.2)	0 (0)	8 (47.1)	28 (87.5)	18 (72.0)	4 (100)	1 (100)	60
Negative	44 (97.8)	18 (100)	9 (52.9)	4 (12.5)	7 (28.0)	0 (0)	0 (0)	82

Total no.	45	18	17	32	25	4	1	142

HPV16 E6 serology								
Seropositive	0 (0)	NA	12 (70.6)	17 (53.1)	11 (44.0)	3 (75.0)	0 (0)	43
Seronegative	45 (100)	NA	5 (29.4)	15 (46.9)	14 (56.0)	1 (25.0)	1 (100)	81

Total no.	45	NA	17	32	25	4	1	124

a Serology data were not available (NA) for the CIN3 patients (*n* = 18).

As shown by the results in Table 1, HPV16 ctDNA was detected at higher levels in samples from patients with tumor stages T2 to T4 than in samples from those with stage T1 (82.0% versus 47.1%, *P* = 0.009). One half of the patients with stage T1 were negative for HPV16 ctDNA, while this biomarker was found in 87.5%, 72.0%, and 100% of patients with stages T2, T3, and T4, respectively.

Multiple HR-HPV infections were found in five plasma samples from CC patients, three of which were positive for HPV16 and HPV18, one for HPV16 and HPV52, and one for HPV16 and HPV31. However, the HPV16 probe in all five specimens generated the highest fluorescence signal (mean fluorescence intensity [MFI]) value (Table S1). The sensitivity and specificity of the HPV16 ctDNA biomarker for identifying HPV16-positive cervical cancer were 74.7% (95% confidence interval [CI] 64.0 to 83.0) and 97.8% (95% CI, 88.2 to 99.9), respectively.

We reanalyzed by droplet digital PCR (ddPCR) the DNA extracted from plasma samples that were previously tested by E7-MPG. In agreement with the E7-MPG data, the results for 30 samples showed an inadequate amount of DNA, being concordantly beta-globin negative by E7-MPG and ESR1 negative by ddPCR. In addition, 12 samples were positive for the housekeeping gene in only one assay, namely, (i) 7 beta-globin-positive and ESR1-negative samples and (ii) 5 beta-globin-negative and ESR1-positive samples (Table S2).

To compare the performance of the two assays for the detection of HPV16 ctDNA, we considered 135 samples (41 controls, 18 CIN3 cases, and 76 CC cases) that were positive for the housekeeping gene with both assays, and we excluded the 42 samples described above. Altogether, 11 plasma samples that were HPV16 ctDNA positive by E7-MPG were negative by ddPCR (one T1, three T2, five T3, and two T4), and only one stage T2 plasma sample was positive by ddPCR and negative by E7-MPG. The overall agreement between both HPV assays for HPV16 ctDNA detection was 91.1% (κ = 0.82; 95% CI, 0.72 to 0.91) which corresponded to strong agreement (Table 2; Table S2).

**TABLE 2:** Comparison of E7-MPG and ddPCR results

Result in E7-MPG assay	No. (%) with indicated result in ddPCR assay^*a*^	
	Positive	Negative
Positive	48 (35.6)	11 (8.1)^*b*^
Negative	1 (0.7)^*c*^	75 (55.6)

a Number of observed agreements: *n* = 123 (91.1%); κ = 0.82, 95% CI, 0.72 to 0.91.

b T1, *n* = 1; T2, *n* = 3; T3, *n* = 5; and T4, *n* = 2.

c T2, *n* = 1.

HPV16 ctDNA was detected by ddPCR in 36.3% of all extracted plasma specimens (49/135) and in 63.2% (48/76) of CC plasma samples. Of the 48 cases, 32 were classified as early stages (T1, *n* = 6, and T2, *n* = 26), while 15 were classified as advanced stages (T3, *n* = 13, and T4, *n* = 2) and one case was classified as an unknown T stage. A quantitative analysis of HPV16 ctDNA-positive specimens showed that the mean HPV16 ctDNA level was higher, though not to a statistically significant level, in advanced T stages (2,362.1 copies/mL) than in early stages T2 (1,346.2 copies/mL; *P* = 0.88) and T1 (251.7 copies/mL; *P* = 0.61).

Similar to the results generated by the E7-MPG, none of the CIN3 plasma samples was positive for HPV16 ctDNA and one control sample was positive for ctHPV16 DNA (Table S2).

In order to evaluate the sensitivity of both assays, serial dilutions of an HPV16 DNA plasmid diluted in genomic DNA were used as the template (from 1,000 to 0 copies of the viral genome). Positive signals were obtained by E7-MPG and ddPCR down to one copy of the viral genome.

Next, HPV16 ctDNA positivity in plasma samples analyzed by the E7-MPG was compared to the previously published serology data regarding E6 antibodies (27). In our study, HPV16 E6 antibodies were detected in 34.7% (*n* = 43) of all available plasma specimens (*n* = 124). Of the 43 cases, 12 were classified as stage T1, 17 as stage T2, 11 as advanced or stage T3, and 3 as stage T4 (Table 1). All controls were HPV16 E6 seronegative (Table 1). The sensitivity and specificity of the HPV16 E6 antibodies for identifying HPV16-positive cervical cancer in this set of samples were 54.4% (43/79) (95% CI, 43.5 to 64.9) and 100% (95% CI, 90.6 to 100), respectively. Moreover, one control specimen tested positive for both HPV18 ctDNA and HPV18 E6 serology (Table S1).

Of the 59 ctHPV16 DNA-positive CC samples, 34 (57.6%) were seropositive for E6, while 25 (42.4%) were seronegative for E6 (Table 2). Of the 20 HPV16 ctDNA-negative CC cases, 9 were seropositive for E6, while 11 were seronegative (3 in stage T1, 2 in T2, and 6 in T3). Among the controls, 44/45 were negative for both HPV16 ctDNA and E6 serology, while 11 CC cases (3 in stage T1, 2 in T2, and 6 in T3) were negative for both markers (Table 3). The use of both HPV16 ctDNA and E6 antibodies for HPV16-positive CC detection improved sensitivity to 86.1% (95% CI, 76.5 to 92.2), while the reported specificity was 97.8% (95% CI, 87.3 to 99.9). Using this combination of HPV markers, the sensitivities for the detection of CCs in stages T1, T2, T3, and T4 were 82.4% (*n* = 14/17), 93.8% (*n* = 30/32), 76.0% (*n* = 19/25), and 100% (4/4), respectively. Thus, testing HPV16 ctDNA and HPV16 E6 antibodies in combination improved the sensitivity for the detection of CC at early T stages (Tables 1 and 3).

**TABLE 3:** Biomarker combinations using both detection of HPV16 ctDNA by the E7-MPG assay and HPV16 E6 serology

Biomarker combination	No. (%) of participants who were:						
	Controls	Patients with cancer stage:					Total no.
		T1	T2	T3	T4	Unknown T	
HPV16 ctDNA positive and E6 seropositive	0 (0)	6 (35.3)	15 (46.9)	10 (40.0)	3 (75.0)	0 (0)	34 (27.4)
HPV16 ctDNA positive and E6 seronegative	1 (2.2)	2 (11.8)	13 (40.6)	8 (32.0)	1 (25.0)	1 (100)	26 (21.0)
HPV16 ctDNA negative and E6 seropositive	0 (0)	6 (35.3)	2 (6.3)	1 (4.0)	0 (0)	0 (0)	9 (7.2)
HPV16 ctDNA negative and E6 seronegative	44 (97.8)	3 (17.6)	2 (6.3)	6 (24.0)	0 (0)	0 (0)	55 (44.4)

Total no.	45	17	32	25	4	1	124

## DISCUSSION

Blood biomarkers like HPV16 ctDNA, in addition to HPV16 E6 and E7 antibodies, have been evaluated as diagnostic and prognostic markers of HPV-related cancers. The detection of HPV ctDNA in CC has been performed using different molecular assays, including ddPCR, which represents a well validated method for detecting and quantifying small amounts of HPV DNA (28–30). Initial findings indicate that the detection of HPV ctDNA in CC patients has high specificity but modest sensitivity, being reported in advanced disease stages, cervical cancer recurrence, and metastasis (29, 31). However, it is not clear yet whether the use of alternative assays or the combination of several blood biomarkers could improve early diagnosis of CC (15, 32, 33). In this study, we validated the performance of a Luminex-based HPV assay (the E7-MPG) and compared it to ddPCR for the detection of HPV16 ctDNA in archived plasma samples collected from patients with premalignant and malignant cervical lesions. The comparison between the E7-MPG and ddPCR assays showed a strong agreement (κ = 0.82). However, the sensitivity of the E7-MPG test for HPV16-positive CC detection appears to be higher than that of ddPCR (74.7% versus 63.2%; *P* < 0.001), with the main advantage being the ability to perform a multiplex analysis in a single reaction. However, the Luminex technology provides a semiquantitative measurement of HPV16 ctDNA, as opposed to ddPCR, which allows an absolute and therefore precise quantification of the target. About 25% and 36% of the CC specimens tested negative for HPV16 ctDNA by the E7-MPG and ddPCR assays, respectively. These data can be partially explained by the low and therefore undetectable levels of viral DNA released in the bloodstream when tumors are small. Indeed, one half of patients with a stage T1 cancer were negative for HPV16 ctDNA by both HPV ctDNA assays. Moreover, the quality of samples that were archived material may have also negatively impacted the sensitivity of both HPV ctDNA assays.

The E7-MPG analysis showed that HPV16 ctDNA was identified in most CC patients, 74.7% (59/79), while this biomarker was not detected in premalignant lesions (CIN3) by either assay, which is in line with previous studies (21, 22, 29). Thus, HPV16 ctDNA showed a specificity of 97.8% and, at the lowest, a sensitivity of 74.7% for identifying HPV16-positive cervical cancer in the analyzed group. Our findings highlight the limitations of HPV ctDNA detection for the identification of patients with cervical premalignant lesions. Indeed, all CINs analyzed in the study were negative for HPV16 ctDNA by both the E7-MPG and the ddPCR assay, viral DNA being undetectable or absent in the bloodstream, as described elsewhere (21, 22, 29, 34). Thus, the use of such a marker of HPV infection does not constitute a method of choice for clinicians to detect premalignant lesions. Screening with Pap and/or HPV tests remains the best strategy for cervical cancer screening. Although the value of HPV ctDNA in diagnosing cervical premalignant lesions is limited, we believe that this biomarker might nonetheless have the potential to be applied in disease monitoring following the diagnosis of cancer, as shown elsewhere (32, 35, 36). However, the retrospective design of our study did not include the collection of samples at different time points and, therefore, does not allow the evaluation of this biomarker in disease monitoring.

Of note, two controls tested positive for ctDNA from HPV16 and HPV18, respectively, the latter being confirmed by E6 serology, which may indicate unreported or unidentified HPV-related malignancies. Moreover, the ctHPV16 DNA positivity was confirmed by the ddPCR assay.

As mentioned above, E6 and E7 antibodies, though mainly E6, are promising markers for the detection of HPV-associated cancers, mainly for oropharyngeal cancers (OPCs). In oropharyngeal cancers, antibodies against E6 are considered early markers of HPV infection, being detected even a decade before the clinical diagnosis (37–39). In contrast, they are rarely detected in individuals without malignancy (40, 41). In CC, HPV16 early protein antibodies appeared to be late markers (27, 37, 42, 43). Here, we combined the available data for HPV16 E6 antibody seroprevalence with the presence of HPV16 ctDNA in plasma samples. While the sensitivities of E6 antibodies and HPV16 ctDNA for identifying HPV16-positive cervical cancer were 54.4% and 74.7%, respectively, when taken independently, the use of both E6 antibodies and HPV16 ctDNA appeared to be a promising marker combination, with a sensitivity of 86.1% (95% CI, 76.5 to 92.2) and specificity of 97.8% (95% CI, 87.3 to 99.9) for identifying HPV16-positive cervical cancer.

A key limitation of this study is the use of archived plasma samples obtained from 1985 to 1999, which most likely affected the DNA quality, as shown by the high level of beta-globin-negative samples. We thus believe that the sensitivity for identifying HPV16-positive cervical cancer could be greatly improved by the use of newly collected plasma samples following the most recent recommendations (44). A limited sample size, the lack of serology data for patients with premalignant cervical lesions, and a focus on only HPV16-positive samples constitute other limitations of this proof-of-principle study.

In conclusion, we describe an alternative to ddPCR that has the great advantage of combining multiplex analysis in a single reaction, thus facilitating its use in the routine clinical setting. However, the use of HPV16 ctDNA as a unique biomarker for cervical cancer screening is rather limited due to a lack of sensitivity for earlier T stages, such as T1, and for premalignant lesions (CIN3). Our preliminary data suggest that HPV markers tested in combination (HPV16 ctDNA and HPV16 E6 antibodies) may improve the sensitivity for the detection of CC at early T stages. Therefore, this combination requires validation in larger prospective studies, as well as translation to other HPV-related cancers, such as head and neck and anal cancers, introducing the concept of combined universal markers for early diagnosis and for monitoring after treatment for all HPV-related cancers.

## MATERIALS AND METHODS

### Participants, specimens, and clinical data.

In previous studies, peripheral blood samples were collected from women diagnosed with HPV16-associated CCs and CIN3 lesions and from HPV-negative women with a negative cytology who were enrolled in Algeria, India, Spain, and Colombia (45–48). These studies were part of an international case-control study of invasive CC and HPV coordinated by the International Agency for Research on Cancer (IARC). Inclusion criteria for CC cases were histologic confirmation of CC diagnosis, no previous treatment for CC, and lack of physical or mental impairments that would have made the interview impossible. For control women, exclusion criteria were diagnosis of anogenital tract cancers, cancers of the breast, endometrium, ovary, or colon, benign genital tumors, tobacco-related diseases (i.e., chronic bronchitis or cancers of the head and neck, lung, or bladder), history of hysterectomy or cervical conization, and physical or mental problems. In the serology study (27), all patients with a valid cervical sample for HPV DNA detection (i.e., beta-globin DNA positive) and an available blood sample were selected.

Among archival specimens with a sufficient amount of plasma (≥500 μl), we randomly selected, using the MS Excel RAND function, 100 HPV16-positive samples from 179 available samples (Algeria and India) from patients with cervical squamous cell carcinoma (CC), 20 HPV16-positive specimens from 127 available samples (Spain and Colombia) from patients with CIN3 lesions, and 60 HPV-negative controls among a total of 258 available HPV16-negative and cytology-negative plasma samples from the Algeria and India case-control HPV study. No other criteria of selection were applied. Plasma samples were collected from 1985 to 1999 and stored at −20°C (Algeria and India) or at −40°C (Spain and Colombia). HPV genotyping was performed in previous studies from cervical scrapes using two different assays, namely, GP5+/6+-based PCR followed by hybridization of PCR products in an enzyme immunoassay (EIA) using HPV type-specific (i.e., HPV6, -11, -16, -18, -31, -33, -34, -35, -39, -40, -42, -43, -44, -45, -51, -52, -54, -56, and -58) oligonucleotide probes for the Indian and Algerian cohorts (45, 46) and L1-based PCR and hybridization with probes to detect HPV6, -11, -16, -18, -31, and -33 and beta-globin for the cohorts from Spain and Colombia (47, 48).

Among control women, 14% had neoplastic diseases (i.e., neoplasms of the skin, thyroid, and hemolymphopoietic tissue). The rest were mainly represented by patients admitted for musculoskeletal diseases and traumas.

### ctDNA extraction.

Circulating tumor DNA (ctDNA) was extracted from 500-μl to 1-mL amounts of plasma samples using the QIAamp circulating nucleic acid kit (Qiagen, Hilden, Germany) according to the instructions of the manufacturer. Extracted ctDNA was eluted into a 60-μL final elution volume, quantified using a Qubit fluorimeter and the Qubit double-stranded DNA (dsDNA) HS assay (Thermo Fisher), and stored at −80°C until analysis.

### HPV ctDNA Luminex analysis.

Extracted ctDNA was then analyzed using a well validated HPV type-specific multiplex genotyping test (the E7-MPG) (25, 26). The analysis combines multiplex PCR and bead-based Luminex technology (Luminex Corp., Austin, TX, USA), as previously reported (25). Briefly, a maximum volume of 10 μl of DNA extract was used as the template to perform a single PCR amplification using multiplex HPV type-specific primers that target E7 gene fragments of HPV16 (91 bp) and other mucosal alpha-HPV types (HPV6, -11, -16, -18, -26, -31, -33, -35, -39, -45, -51, -52, -53, -56, -58, -59, -66, -68, -70, -73 and -82) (25, 49). The E7-MPG assay includes an additional two primers for beta-globin PCR amplification as an internal control. After PCR amplification, 10 μl of each reaction mixture was analyzed by using a Luminex-based assay as previously described (25). For each probe, the mean fluorescence intensity (MFI) value obtained when no PCR product was added to the hybridization mixture was considered the background value. The cutoff was computed by adding 5 MFI to 1.1× the median background value. All MFI values above the cutoff were considered positive (25).

### ddPCR.

Validated primers and probe targeting part of the E7 gene (75 bp) were used for the amplification and identification of HPV16. Primer and probe sequences can be found elsewhere (17, 50). In the same reaction mixture, primers and probe from Bio-Rad (Bio-Rad Laboratories, Pleasanton, CA, USA) targeting a region within the ESR1 gene (66 bp) were used for assessing DNA quality (assay identification number dHsaCP1000403). Briefly, in each reaction mixture, 11 μl of 2× droplet digital PCR (ddPCR) supermix for probes (no dUTP) (Bio-Rad), 1 μl of each of the respective primers and probes, and a maximum volume of 9 μl of extracted ctDNA were used in a final volume of 22 μl, of which 20 μl was used for droplet generation. Extracted DNA from human cell lines CaSki (HPV16 positive) and HeLa (HPV18 positive) were used as the positive and negative control, respectively, for HPV16 DNA detection. In addition, to determine the sensitivity of the ddPCR assay, we used serial dilutions of HPV16 DNA diluted in genomic DNA (from 1,000 to 0 copies of the viral genome). Each dilution was tested in duplicate. Then, ddPCR was performed on the QX200 AutoDG droplet digital PCR system platform (Bio-Rad) and analyzed using QuantaSoft Analysis Pro software (Bio-Rad). Samples were considered HPV positive when one or more positive droplets were obtained. No positive droplets were generated in HPV16-negative controls.

### Serology test.

The serological assay was performed at the German Cancer Research Center (DKFZ), Heidelberg, Germany. Briefly, the multiplex HPV serology assay was based on a glutathione *S*-transferase (GST) capture enzyme-linked immunosorbent assay (ELISA) (51, 52) in combination with different fluorescence-labeled polystyrene beads (SeroMAP microspheres; Luminex) carrying several HPV antigens (53, 54). Labeled beads were then incubated with human serum diluted (1:100) in blocking buffer. Antibodies linked to the beads were then marked with biotinylated anti-human IgG and fluorescent streptavidin–*R*-phycoerythrin, used as the conjugate. All details regarding the serology assay were described previously (27). Briefly, antibodies against a broad range of proteins from HPV16 (L1, E1, E2, E4, E6, and E7) and HPV18, -31, -33, -35, -45, -52, and -58 (L1, E6, and E7) were evaluated in two case control studies of cervical cancer in Algeria and India. The authors showed that, of the immune markers tested, E6 and E7 best discriminated CC from controls; however, the most discriminant marker was HPV16 E6. However, CIN3 samples were not analyzed by the serological assay in the previous studies.

The standard cutoff for HPV16 E6 seropositivity of 484 MFI was used (40, 42, 55).

### Statistical analysis.

HPV ctDNA prevalence was estimated as the proportion of plasma samples that tested positive for HPV16 ctDNA by the E7-MPG or ddPCR assay with corresponding binomial 95% confidence intervals (CIs). The performance of each biomarker in the detection of CC, as measured by sensitivity and specificity, was evaluated using the results obtained from cervical samples as the reference (45–48). The Cohen’s kappa coefficient with 95% CI was calculated to estimate the concordance of HPV16 ctDNA detection between the E7-MPG and ddPCR assays. McHugh’s interpretation of the Cohen’s kappa index was used to assess the level of agreement between both HPV assays for HPV16 detection (0 to 0.2, no agreement; 0.21 to 0.39, minimal agreement; 0.40 to 0.59, weak agreement; 0.60 to 0.79, moderate agreement; 0.80 to 0.90, strong agreement; and κ > 0.9, almost perfect agreement) (56).

### Ethics approval.

The study from Algeria was approved by the ethics review committees of the CMPC in Algiers and the IARC in Lyon, and all participants, both CC cases and controls, signed an informed consent form. The Indian study was approved by the ethical review committees of the IARC in Lyon and the Cancer Institute in Chennai, and the study in Spain/Colombia was approved by both IARC and local ethic committees.

### Data availability.

All data generated for this study are included in this published article and its supplemental materials.
